# Seroprevalence of porcine coronavirus antibodies in Iberian pigs and wild boars from central-western Spain

**DOI:** 10.1186/s40813-026-00488-3

**Published:** 2026-02-03

**Authors:** Paloma Encinas, Gustavo del Real, Ronaldo Magtoto, Juan Carlos Mora-Díaz, Lu Yen, María Templado, Irene Iglesias, José A. Carrillo-Ávila, Jesús Hernández, Luis G. Giménez-Lirola

**Affiliations:** 1 Department of Biotechnology, National Center Institute for Agricultural and Food Research and Technology (INIA-CSIC), Madrid, Spain; 2 Department of Veterinary Diagnostic and Production Animal Medicine, College of Veterinary Medicine, Iowa State University, Ames, IA USA; 3 Animal Health Research Centre (CISA), National Centre Institute for Agriculture and Food Research and Technology (INIA-CSIC), Madrid, Spain; 4 Andalusian Public Health System Biobank, Granada, Spain; 5 Laboratory of Immunology, Centro de Investigación en Alimentación y Desarrollo, Hermosillo, Sonora, Mexico

**Keywords:** Porcine coronavirus, Iberian pigs, Wild boars, Seroprevalence, Free-range pigs, PHEV, PEDV, PDCoV, PRCV, TGEV

## Abstract

Porcine coronaviruses (PoCoVs) are common etiological viral agents of enteric and respiratory disease in swine, but most epidemiological information derives from intensively managed herds. Data from outdoor systems and wildlife remain scarce, despite the potential role of free-range production and wildlife-livestock interactions in sustaining virus transmission. In southern Spain, the traditional Dehesa agroforestry system supports Iberian pigs that share space and resources with wild boars and other species, creating interfaces where cross-species circulation may occur. To address this gap, we assessed the seroprevalence of five PoCoVs, including porcine hemagglutinating encephalomyelitis virus (PHEV), porcine epidemic diarrhea virus (PEDV), porcine deltacoronavirus (PDCoV), transmissible gastroenteritis virus (TGEV), and porcine respiratory coronavirus (PRCV), in Iberian pigs and wild boars from central-western Spain. A total of 260 Iberian pig sera and 564 wild boar sera collected between 2016 and 2020 were tested using indirect ELISAs for PHEV, PEDV, and PDCoV and a blocking ELISA for PRCV/TGEV. Antibodies to PHEV were highly prevalent in Iberian pigs (68.0%) and detected at lower levels in wild boars (22.6%), a pattern consistent with endemic exposure in domestic pigs and sporadic circulation in wildlife. PEDV antibodies were identified in 8.5% of Iberian pigs and 2.8% of wild boars, with higher prevalence in pigs during 2016 followed by a sharp decline, suggesting past but not ongoing activity. PDCoV antibodies were rare overall (3.1% in pigs, 2.6% in wild boars) but reached 41.6% in pigs from Cáceres in 2017-2018, indicative of a localized event. PRCV antibodies were widespread in Iberian pigs (67.3%), with higher prevalence in Badajoz compared to Cáceres, while wild boars showed rising seropositivity in Ciudad Real/Toledo by 2022 (up to 33.3%). No TGEV antibodies were detected in either host population, supporting the predominance of PRCV in the region. These findings demonstrate that PHEV and PRCV are enzootic in free-range Iberian pigs, while PEDV and PDCoV circulate at low levels, and wild boars are more likely incidental than reservoir hosts. The detection of antibodies in the absence of clinical outbreaks underscores the silent nature of PoCoV circulation in extensive systems and highlights the importance of integrating wildlife-livestock interfaces into surveillance and biosecurity strategies in Mediterranean production landscapes.

## Introduction

Coronaviruses (CoVs) are enveloped, positive-sense single-stranded RNA viruses with high mutation and recombination rates, facilitating adaption to new hosts and ecological niches [1]. Within the subfamily *Orthocoronavirinae*, porcine coronaviruses (PoCoVs) are classified into three genera: *Alphacoronavirus* (which includes transmissible gastroenteritis virus [TGEV], porcine respiratory coronavirus [PRCV], porcine epidemic diarrhea virus [PEDV], and swine acute diarrhea syndrome coronavirus [SADS-CoV], *Betacoronavirus* (porcine hemagglutinating encephalomyelitis virus [PHEV], and *Deltacoronavirus* (porcine deltacoronavirus [PDCoV] [1–3].

These viruses infect the respiratory and/or gastrointestinal tract of pigs and are responsible for enteric and respiratory diseases of variable severity [2, 4]. Enteric PoCoVs such as TGEV, PEDV, SADS-CoV, and PDCoV cause acute diarrhea, dehydration, growth retardation, and high mortality in neonatal pigs and are considered major threats to swine health and production [4, 5]. PHEV, though less frequently reported, causes vomiting, wasting, and encephalomyelitis primarily in piglets under four weeks of age. However, like other PoCoVs, it is often subclinical in older pigs or immune herds, where prior exposure or maternal immunity mitigates clinical signs [4, 6]. PRCV, a naturally occurring deletion mutant of TGEV identified in the 1980s [7, 8], shows a shift in tropism toward the respiratory tract but elicits cross-reactive and cross-protective antibodies indistinguishable from TGEV by conventional serological tests [4, 7]. SADS-CoV has been identified in Asia but has not been reported in Europe nor USA to date [9, 10].

PoCoV surveillance and field research have predominantly focused on pigs raised under intensive confinement, where outbreaks are economically devastating and biosecurity is tightly regulated. In contrast, far less is known about virus circulation in alternative production systems (e.g., free-range pigs) or wildlife. This knowledge gap is particularly relevant in southern Europe, where traditional extensive farming systems such as the *Dehesas* predominates. The *Dehesa* is a multifunctional agroforestry system characterized by grassland interspersed with low-density *Quercus* spp. trees. These systems support a mosaic land uses, including the outdoor rearing of Iberian pigs with minimal confinement [11]. Animals often share space and resources with livestock (e.g., cattle, sheep, and goats) and wildlife species such as red deer (*Cervus elaphus*) and wild boars (*Sus scrofa*) [12]. This complex ecological interface facilitates direct or indirect interactions across species, potentially increasing the risk of cross-species transmission of pathogens, including porcine PoCoV [13–18]. Wild boars, which are widespread across the Iberian Peninsula, are susceptible to PoCoV and may act as reservoirs or spillover hosts [18, 19]. Bat-like CoVs in swine population have been detected in pigs in the Iberian Peninsula underscoring the potential risk of interspecies spillover [20].

Serological studies have documented PoCoV exposure in the wild and extensively raised pigs in several regions, although data remain limited. In Spain, available information is restricted to conventional indoor systems: a nationwide study reported a 29.5% seroprevalence for PEDV in indoor-bred domestic pigs [21], but no data exist for wild boars or Iberian pigs raised outdoors. Elsewhere in Europe, antibodies to TGEV or PRCV have been detected in wild boars from Poland (3.2%), Slovenia (3.0% PRCV, none for TGEV), and Italy, where retrospective testing revealed seropositivity for PEDV (3.83%) and TGEV/PRCV (0.67%) [16, 19, 22, 23]. In South America, 3.4% of wild boars tested in Argentina were seropositive for TGEV [18]. In the U.S., PEDV antibodies were detected in 3.2% of feral pigs by enzyme-linked immunosorbent assay (ELISA), although confirmatory neutralization assays indicated a much lower rate of true exposure (0.1%), suggesting that some ELISA-positive results may represent false positives [24]. Widespread subclinical circulation of PHEV has also been documented in U.S. breeding herds, with antibodies detected in 53.3% of sows [6]. In Asia, PEDV and PDCoV are enzootic, with seroprevalence often exceeding 50% during outbreak periods [25–27]. SADS-CoV has also caused severe outbreaks in neonatal pigs in southern China but has not been reported outside the region [9, 10].

Despite growing international evidence of PoCoV circulation in both commercial and free-ranging pigs [2, 28], no studies have evaluated the seroprevalence of these viruses in Iberian pigs or wild boars in Spain. The objective of this study was to assess the presence of antibodies against five major PoCoVs (i.e., TGEV, PRCV, PHEV, PEDV, and PDCoV) in populations of free-range Iberian pigs and wild boars in the southwestern regions of Spain, particularly in areas of Andalucía and Extremadura where extensive Dehesa-based pig farming is practiced. The results contribute to a broader understanding of PoCoV ecology in outdoor swine production systems and wildlife interfaces in Europe.

## Methods

### Sample collection

**Fig. 1:**
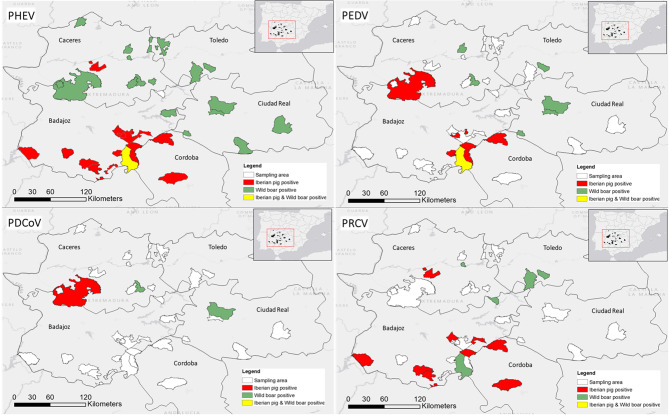
Geographical distribution of sampling sites included in the seroprevalence survey for porcine coronaviruses (PoCoV) in central-western Spain. Map depicting the four provinces, i.e., Cáceres, Toledo, Badajoz, and Ciudad Real, where serum samples from Iberian pigs and wild boars were collected for the detection of antibodies against porcine hemagglutinating encephalomyelitis virus (PHEV), porcine epidemic diarrhea virus (PEDV), porcine deltacoronavirus (PDCoV), transmissible gastroenteritis virus (TGEV), and porcine respiratory coronavirus (PRCV). The red square on the inset map of Spain (bottom left) highlights the location of the study area within the national context

Serum samples were collected from free-ranging domestic Iberian pigs and wild boars in Central-Western Spain between 2016 and 2020 (Fig. 1). The study area encompassed the Extremadura region (provinces of Cáceres and Badajoz) and the Castilla-La Mancha region (provinces of Toledo and Ciudad Real), covering approximately 76,900 km^2^ (Fig. 1). This territory includes forested mountain areas, traditional *Dehesa* landscapes, and extensive agricultural plains dedicated to cereal and wine production. The four provinces support large populations of wild boars and numerous hunting reserves, while Cáceres y Badajoz are home to a high density of free-range Iberian pig farms, creating potential interfaces for direct or indirect interaction between domestic and wild suids.

Opportunistic wild boar serum samples (*n* = 564) were obtained from two sources: legally hunted animals during the hunting season (October-March), and live-captured animals sampled as part of routine active health surveillance programs. These samples were collected from 33 herds across 11 municipalities in Extremadura and from 41 herds across 8 municipalities in Castilla-La Mancha (i.e., Toledo and Ciudad Real)

Serum samples from Iberian pigs (*n* = 260) were collected at slaughterhouses during routine abattoir operations. These pigs originated from 26 herds in 19 municipalities across the Extremadura provinces of Cáceres and Badajoz. In both populations, animals were selected randomly regardless of sex or age and included both juveniles and adults.

All sampled Iberian pigs and wild boars were clinically healthy at the time of collection, and no clinical signs compatible with coronavirus infection were observed or recorded during routine slaughterhouse inspection, hunting activities, or wildlife surveillance procedures.

All sampling activities were conducted in accordance with Spanish national legislation. Samples were obtained from animals that were legally hunted, captured, or slaughtered for commercial purposes; therefore, formal ethical approval by an institutional animal care and use committee was not required. Blood samples from hunted wild boars were collected via standardized cavernous sinus puncture [29]. After clotting, sera were separated and stored frozen at -20 °C until analysis. Prior to serological testing, all samples were heat-inactivated at 55 °C for 30 min to reduce non-specific binding in the assays.

### ELISA

Serum samples were tested for antibodies against PHEV, PEDV, PDCoV, and TGEV/PRCV using indirect or blocking ELISAs. In-house indirect ELISAs targeting the S1 domain of the spike (S) protein were used for PHEV, PEDV, and PDCoV and cutoffs were selected to ensure 100% diagnostic specificity. The antigens and protocols for PHEV and PDCoV were previously described and validated [30, 31]. The S1 protein used for PEDV was produced and evaluated for diagnostic performance using fluorescent microsphere-based immunoassay (FMIA) in an earlier study [32]; for the present work, this antigen was adapted to the indirect ELISA format following the same procedures described for PHEV [30]. A commercial blocking ELISA (INGEZIM Coronavirus, Ingenasa/Gold Standard Diagnostics, Madrid, Spain), previously evaluated for field performance [33], was used for detecting anti-TGEV/PRCV antibodies following manufacturer’s instructions for testing conditions and result interpretation.

All ELISA tests were performed in duplicate on 96-well plates with appropriate positive and negative controls. Interpretation was based on the established cutoff criteria for each assay (sample-to-positive [S/P] ratio or percent inhibition [PI]). Complete assay details, including antigen target, incubation conditions, cutoff values, and relevant references are summarized in Table 1.

**Table 1:** Summary of the ELISAs used for detection of anti-porcine coronavirus antibodies in serum from Iberian pigs and wild boars

Virus	ELISA type	Antigen/Antibody used	Incubation conditions	Sample dilution	Cutoff criteria	Reference
PHEV	Indirect ELISA	Recombinant S1 protein	1 h at 37 °C (antigen and serum); 30 min at 37 °C (conjugate); 5 min at RT^1^ (substrate)	1:100	S/*P* > 0.6 (positive)	Mora-Díaz et al., 2020
PEDV	Indirect ELISA	Recombinant S1 protein	1 h at 37 °C (antigen and serum); 1 h at 37 °C (conjugate); 5 min at RT (substrate)	1:100	S/*P* > 0.6 (positive)	Giménez-Lirola et al., 2016^2^
PDCoV	Indirect ELISA	Recombinant S1 protein	1 h at 37 °C (antigen and serum); 30 min at 37 °C (conjugate); 5 min at RT (substrate)	1:100	S/*P* > 0.25 (positive)	Yen et al., 2022
TGEV/PRCV	Blocking ELISA	TGEV S protein and TEGV/PRCV monoclonal antibodies	1 h at 37 °C (antigen and serum); 30 min at 37 °C (conjugates); 10 min at RT (substrate)	1:1	Interpretation followed the manufacturer’s guidelines (Ingenasa, Ref: 11.DIF.K3).	Magtoto et al., 2019

^1^RT; room temperature (20–24 °C). ^2^The diagnostic performance of the PEDV S1 recombinant protein was originally assessed via indirect fluorescent microsphere-based immunoassay (FMIA; reference provided). In the present study the protein was used as antigen in an indirect ELISA format, following protocols previously described for the PDCoV and PHEV indirect ELISAs

### Statistical analysis

The data were analyzed using the Shapiro-Wilk and Kolmogorov-Smirnov tests to evaluate normality, and as a result, a non-parametric approach was applied for further analysis. Differences in antibodies between Iberian pigs and wild pigs were evaluated using the Mann-Whitney test. All analyses were conducted with a significance level > 0.05 using GraphPad Prism software version 10.4.0 (GraphPad Software, Boston, MA, USA).

## Results

### Prevalence of antibodies against PHEV

**Fig. 2:**
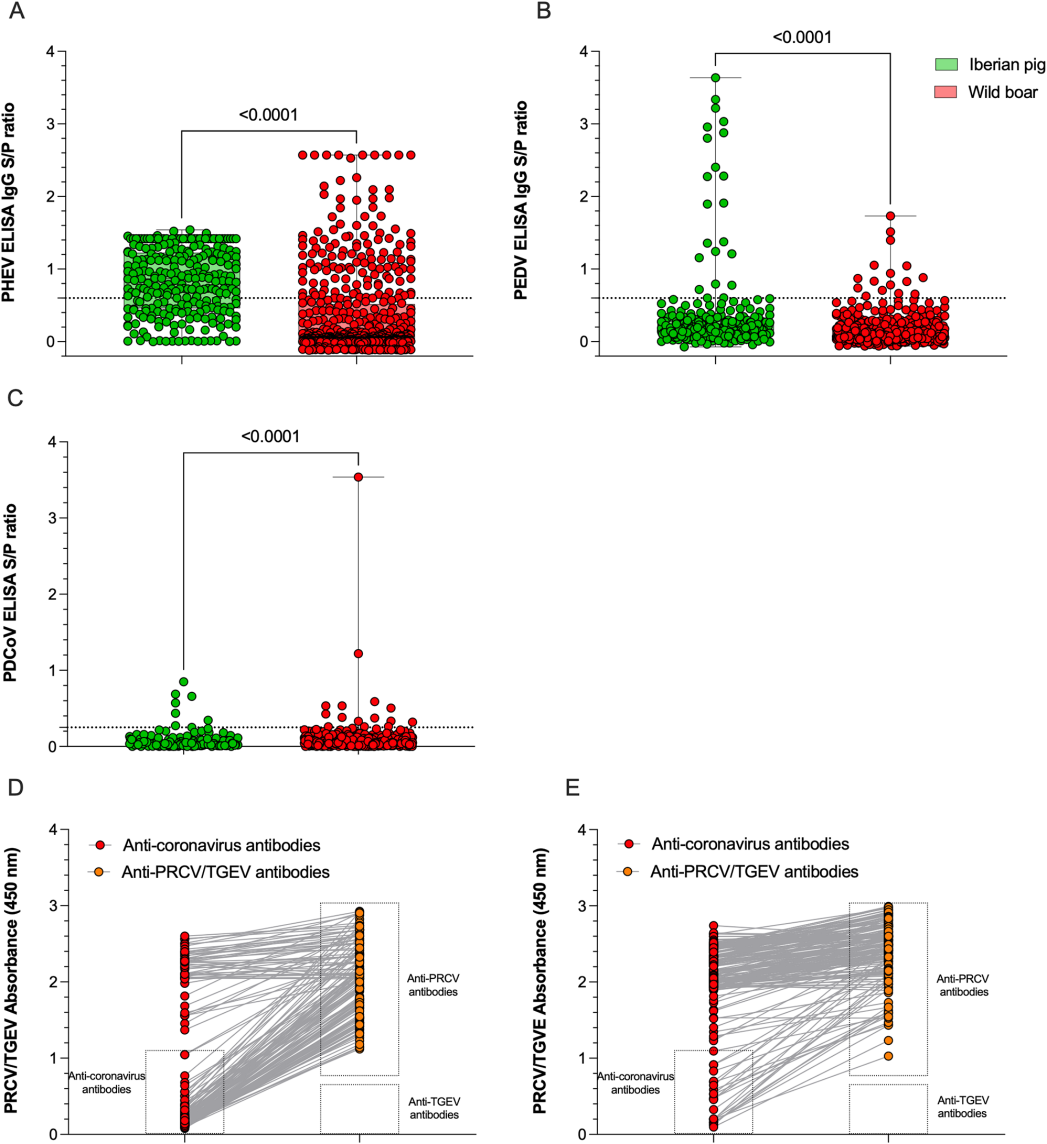
Distribution of ELISA results for porcine coronaviruses (PoCoV) in Iberian pigs and wild boars. (A-C) Sample-to-positive (S/P) ratios obtained using indirect ELISAs for detection of antibodies against porcine hemagglutinating encephalomyelitis virus (PHEV, A), porcine epidemic diarrhea virus (PEDV, B), and porcine deltacoronavirus (PDCoV, C) in Iberian pigs (green) and wild boars (red). Assays were previously validated and based on recombinant spike S1 antigens. Dotted lines indicate assay-specific thresholds for seropositivity. Statistical comparisons between host species were performed using the Mann–Whitney test (*p* < 0.0001 for all panels). (D-E) Absorbance values (OD450) obtained from a commercial blocking ELISA used to differentiate antibodies to transmissible gastroenteritis virus (TGEV) and porcine respiratory coronavirus (PRCV) in Iberian pigs (D) and wild boars (E). Each line represents an individual sample tested for both antibody specificities. Gray shaded boxes denote positive result areas

Antibodies against PHEV were detected in both Iberian pigs and wild boars; however, ELISA antibody levels were significantly higher in Iberian pigs than in wild boars (*p* < 0.0001; Fig. 2). The overall seroprevalence in Iberian pigs was 68.0% (176/259), notably higher than in wild boars, where 22.6% (127/562) of animals tested positive (Fig. 2). Among Iberian pigs, seroprevalence was higher in Badajoz (70.9%) compared to Cáceres (50.0%) (Table 2).

**Table 2:** Porcine coronavirus antibody prevalence in Iberian pigs and wild boars

Year	Iberian pig			Wild boar		
	Badajoz	Cáceres	TOTAL	Badajoz and Cáceres	Ciudad Real and Toledo	TOTAL
	**PHEV**					
2016	56/70 (80,0)	0/0	56/70 (80,0)	ND	ND	
2017/2018	19/24 (79,1)	0/12 (0,0)	19/36 (52,7)	ND	ND	
2019	13/26 (50,0)	13/17 (76,4)	26/43 (60,4)	1/12 (8,3)	3/17 (17,6)	4/29 (13,7)
2020	66/97 (68,1)	9/13 (69,2)	78/110 (70,9)	13/57 (22,8)	0/37 (0)	13/94 (13,8)
2021	ND	ND		18/86 (20,9)	40/176 (22,7)	58/262 (22,1)
2022	ND	ND		18/77 (23,4)	34/100 (34,0)	52/177 (29,3)
Total	159/217 (70,9)	21/42 (50,0)	179/259 (69,1)	50/232 (21,5)	77/330 (26,0)	127/562 (22,5)
	**PEDV**					
2016	17/70 (24,3)	0/0	17/70 (24,3)	ND	ND	
2017/2018	2/24 (8,3)	1/12 (8,3)	3/36 (8,3)	ND	ND	
2019	0/26 (0,0)	1/17 (5,8)	1/43 (2,3)	0/12 (0,0)	1/17 (5,8)	1/29 (3,4)
2020	1/98 (1,0)	0/13 (0,0)	1/111 (0,9)	2/57 (3,5)	1/37 (2,7)	3/94 (2,1)
2021	ND	ND		2/85 (2,4)	9/178 (5,0)	11/263 (4,1)
2022	ND	ND		0/77 (0,0)	1/100 (1,0)	1/177 (0,5)
Total	20/218 (9,1)	2/42 (4,7)	22/260 (8,5)	4/231 (1,7)	12/332 (3,6)	15/562 (2,8)
	**PDCoV**					
2016	2/70 (2,8)	0/0 (0,0)	2/70 (2,8)	ND	ND	
2017/2018	0/24 (0)	5/12 (41,6)	5/36 (13,8)	ND	ND	
2019	0/26 (0)	1/17 (5,8)	1/43 (2,3)	0/12 (0)	0/17 (0,0)	0/29 (0,0)
2020	0/98 (0)	0/13 (0)	0/111 (0)	2/57 (3,5)	1/37 (2,7)	1/94 (1,0)
2021	ND	ND		1/87 (1,1)	6/178 (3,3)	1/265 (0,3)
2022	ND	ND		1/77 (1,2)	4/100 (4,0)	0/177 (0,0)
Total	2/218 (0,9)	6/42 (14,2)	8/260 (3,0)	4/233 (1,7)	11/332 (0,3)	15/565 (2.6)
	**PRCV**					
2016	ND	ND		ND	ND	
2017/18	ND	ND		ND	ND	
2019	22/26 (84,6)	9/16 (56,2)	31/42 (73,8)	ND	ND	
2020	71/98 (72,4)	1/13 (7,7)	75/111 (64,8)	3/38 (7,8)	1/30 (3,3)	4/68 (5,8)
2021	ND	ND		1/12 (8,3)	3/41 (7,3)	4/53 (7,5))
2022	ND	ND		3/37 (4,1)	12/36 (33,3)	15/73 (20,5)
Total	93/124 (75,0)	10/29 (34,5)	103/153 (67,3)	7/87 (5,7)	16/107 (14,9)	23/194 (10,8)
	**TGEV**					
2016	ND	ND		ND	ND	
2017/18	ND	ND		ND	ND	
2019	0/26 (0,0)	0/16 (0,0)	0/42 (0,0)	ND	ND	
2020	0/98 (0,0)	0/13 (0,0)	0/111 (0,0)	0/38 (0,0)	0/30 (0,0)	0/68 (0,0)
2021	ND	ND		0/12 (0,0)	0/42 (0,0)	0/54 (0,0)
2022	ND	ND		0/37 (0,0)	0/36 (0,0)	0/73 (0,0)
Total	0/124 (0,0)	0/29 (0,0)	0/153 (0,0)	0/87 (0,0)	0/108 (0,0)	0/195 (0,0)

Yearly prevalence in Iberian pigs ranged from 52.7% to 80.0%. In Badajoz, the highest seroprevalence was recorded in 2016 (80.0%), while the lowest was in 2019 (50.0%). In Cáceres, no positive samples were detected in 2017 or 2018; however, seroprevalence increased to 76.4% in 2019 and 69.2% in 2020 (Table 2).

In wild boars, seroprevalence was similar between Badajoz/Cáceres (21.5%) and Ciudad Real/Toledo (26.0%). Annual prevalence in wild boars ranged from 13.0% to 29.0%. In Badajoz/Cáceres, seroprevalence was lowest in 2019 (8.3%) and ranged from 20.9% to 23.4% between 2020 and 2022. In Ciudad Real/Toledo, annual seroprevalence varied more widely, from 0.0% in 2020 to 34.0% in 2022 (Table 2).

### Prevalence of antibodies against PEDV

Serological testing identified antibodies against PEDV in both Iberian pigs and wild boars, though at relatively low levels; antibody levels were significantly lower in wild boars than in Iberian pigs (*p* < 0.0001; Fig. 2). Among Iberian pigs, 8.5% (22/260) of the samples were seropositive (Fig. 2). Regionally, seroprevalence was higher in Badajoz (9.1%) compared to Cáceres (4.7%). Temporal analysis showed the highest positivity in 2016 (24.3%), with a progressive decline in subsequent years, reaching 1.0% by 2020. Notably, all 2016 seropositive Iberian pigs were from Badajoz; in later years, positives were more evenly distributed between the two provinces (Table 2).

In wild boars, overall seroprevalence was lower at 2.8% (16/562). When grouped by region, samples from Ciudad Real and Toledo showed higher seroprevalence (3.6%) compared to those from Badajoz and Cáceres (1.7%). Seroprevalence in wild boars varied modestly across years, with 3.4% in 2019, 2.1% in 2020, and 4.1% in 2021, followed by a marked decrease to 0.5% in 2022. In Badajoz/Cáceres, PEDV-positive wild boar samples were identified only in 2017/2018 and 2019, whereas seropositive individuals from Ciudad Real/Toledo were detected across all years evaluated (Table 2).

### Prevalence of antibodies against PDCoV

Serological evidence of PDCoV exposure was detected at low levels in both Iberian pigs and wild boars; however, ELISA antibody levels were significantly higher in wild boars than in Iberian pigs (*p* < 0.0001; Fig. 2). Despite this difference, overall seroprevalence was comparable between the two groups, with 3.1% (8/260) of Iberian pig sera and 2.6% (15/565) of wild boar sera testing positive for anti-PDCoV antibodies (Fig. 2).

In Iberian pigs, PDCoV seroprevalence was markedly higher in Cáceres (14.2%) compared to Badajoz (0.9%). When analyzed by year and region, 13.8% of samples collected in Cáceres in 2017/2018 were seropositive, while lower rates were observed in 2016 (2.3%) and 2019 (2.8%). No seropositive animals were detected in 2020. In Badajoz, only samples from 2016 were tested positive (2.8%), with no evidence of PDCoV antibodies in subsequent years.

In wild boars, PDCoV seroprevalence remained consistently low across the study period. No positive samples were detected in 2019. In contrast, 3.1%, 2.6%, and 2.8% of wild boars sampled in 2020, 2021, and 2022, respectively, were seropositive. Regional analysis showed a peak in Badajoz/Cáceres in 2020 (3.5%), while Ciudad Real/Toledo presented relatively stable rates between 2.7% and 4.0% from 2020 to 2022 (Table 2).

### Prevalence of antibodies against the PRCV/TGEV

Serological testing revealed a high seroprevalence of antibodies against PRCV in Iberian pigs, with 67.3% (103/153) samples testing positive. In contrast, only 11.7% (23/195) of wild boar sera were positive for PRCV antibodies. No samples from either population tested positive for TGEV antibodies (Fig. 2).

Among Iberian pigs, only sera collected in 2019 and 2020 were available for PRCV analysis. The seropositivity was 73.8% in 2019 and 64.8% in 2020. Regional analysis showed a notably higher prevalence in Badajoz (75%) compared to Cáceres (34.5%). In Badajoz, the prevalence was relatively stable across years: 84.6% in 2019 and 72.4% in 2020. In contrast, in Cáceres, the prevalence dropped from 56.2% in 2019 to 7.7% in 2020.

In wild boars, PRCV seropositivity was 5.8% in 2020, 5.5% in 2021, and increased to 20.5% in 2022. Regional differences were evident: 5.7% of wild boars from Badajoz/Cáceres were seropositive, while in Ciudad Real/Toledo, the overall prevalence was 14.9%. Within Ciudad Real/Toledo, seroprevalence increased from 0% in 2020 to 33.3% in 2022. In Badajoz/Cáceres, rates remained lower and more stable: 7.8% in 2020, 8.3% in 2021, and 4.1% in 2022 (Table 2).

No evidence of TGEV-specific antibodies was found in any of the 153 Iberian pig or 195 wild boar sera tested (Table 2).

## Discussion

The extensive Dehesa system characteristic of Southwestern Spain provides a unique ecological niche in which domestic free-range pigs, wild boars, and other wildlife species coexist. This sylvopastoral environment, composed of Mediterranean oak woodlands, seasonal pastures, and low animal densities, promotes natural behavior and high-quality meat production. However, it also facilitates pathogen transmission at the wildlife-livestock interface through shared water sources, environmental persistence, and indirect or direct interspecies contact.

While most seroprevalence studies on PoCoVs have focused on intensively reared pigs, our findings indicate that free-range and wild swine populations can also be exposed to multiple PoCoVs. The detection of anti-PoCoVs antibodies in Iberian pigs raised in open systems supports the notion that these viruses can circulate silently in extensive production settings, without overt clinical disease. Subclinical infections are common across PoCoVs, particularly in grower-finisher and adult pigs [4, 30, 33–35] and are consistent with antibody detection in the absence of outbreak history.

### PHEV

The high seroprevalence of PHEV among Iberian pigs (> 67%) mirrors results from North American intensive systems, where widespread subclinical circulation has been reported [6, 30]. The consistent PHEV detection in both Badajoz and Cáceres provinces, and the regional uniformity in wild boars, suggest endemic exposure. However, temporal variability, such as the absence of PHEV seropositive Iberian pigs in Cáceres in 2017/2018 followed by marked increases, may reflect local shifts in virus transmission or immunity. In wild boars, lower and more stable seroprevalence across years supports sporadic exposure without sustained transmission.

### PEDV

The overall low PEDV seroprevalence in both pig populations aligns with prior findings in wild boars across Europe and Asia [16, 22, 23, 36]. The relatively higher seroprevalence in Iberian pigs in 2016 suggests past circulation or spillover from commercial farms, with the subsequent decline over time possibly reflecting improved biosecurity or declining regional virus pressure. The low and sporadic detection of antibodies in wild boars supports their role as incidental hosts rather than competent reservoirs. These findings indicate that PEDV exposure can occur in outdoor systems, albeit at low levels.

It is important to consider that PEDV display considerably genetic and antigenic variability, largely driven by mutations and deletions in the *S* gene. In this study, antibody detection was performed using an indirect ELISA based on a recombinant S1 domain of the PEDV S protein, previously validated for serological detection and shown to capture antibody responses across genetically diverse PEDV strains [32]. Therefore, the detected seropositivity likely reflects prior exposure to antigenically related PEDV strains rather than strain-specific circulation. Serology does not allow discrimination between PEDV genogroups; therefore, molecular characterization would be required to determine the specific strains involved.

### PDCoV

PDCoV seroprevalence was also low but detectable in both populations. Notably, the increase in Cáceres between 2017 and 2018 (41.6%) may reflect a localized outbreak, although the absence of seropositive animals in other years or locations suggests sporadic circulation. The limited seroprevalence across wild boar samples is consistent with rare or indirect exposure, possibly through contaminated environments. Previous studies have reported minimal PDCoV detection in outdoor systems Tang et al. 2021 [25], and our findings support its limited persistence under such conditions.

### PRCV and TGEV

The high PRCV seroprevalence in Iberian pigs, especially in Badajoz, aligns with historical data from intensively managed pigs, where PRCV infection is widespread and typically subclinical. The observed regional variability (e.g., steep decline in Cáceres in 2020) may reflect differences in herd immunity, contact with commercial pigs, and/or other ecological factors influencing transmission. The presence of PRCV antibodies in wild boars, particularly the notable increase in Ciudad Real/Toledo in 2022, suggests episodic spillovers from domestic pigs or exposure to contaminated environments.

PRCV, a spike gene deletion mutant of TGEV [8], elicits cross-reactive and cross-protective antibodies indistinguishable from those of TGEV in standard serological tests [7, 33]. The absence of TGEV-specific antibodies in our study supports the hypothesis that detected antibodies reflect PRCV exposure. This finding is consistent with broader trends in Europe and North America, where PRCV has largely replaced TGEV in domestic herds due to competitive exclusion and changes in vaccine practices [2].

### Implications for surveillance and control

The results of this study highlight the importance of including extensive production systems and wildlife in PoCoV surveillance efforts. Although the clinical impact of PoCoVs in these settings appears limited, the presence of antibodies suggests that viruses can circulate silently. Integrated surveillance approaches, combining serology, virology, and ecological data, will be essential to understanding virus persistence and transmission pathways in agroecological landscapes such as the *Dehesa*.

Despite the large sample size and multi-year scope, the study had limitations, including uneven sample distribution across regions and years, especially among Iberian pigs in Cáceres. Nevertheless, the trends observed were robust and consistent with epidemiological expectations based on virus behavior and environmental context.

## Conclusions

This study provides the first comprehensive serological assessment of five PoCoVs in free-range Iberian pigs and wild boars in southwestern Spain. PHEV and PRCV were the most prevalent, particularly in domestic pigs, while PEDV and PDCoV had low but detectable exposure rates. No serological evidence of TGEV circulation was found. These findings underscore the need to include outdoor systems and wildlife in disease monitoring frameworks to better understand the ecology of PoCoVs and control strategies in diverse production systems.

## Data Availability

The data supporting this study’s findings are available within the article and its supplementary information files (Table Source data).- The datasets used and/or analyses during the current study are available from the corresponding author upon request.- All data generated or analyses during this study are included in this published article.
